# Potential Health-Promoting Benefits of Paraprobiotics, Inactivated Probiotic Cells

**DOI:** 10.4014/jmb.1911.11019

**Published:** 2020-01-09

**Authors:** Shahina Akter, Jong-Hyun Park, Hoo Kil Jung

**Affiliations:** Department of Food Science and Biotechnology, College of BioNano Technology, Gachon University, Seongnam 13120, Republic of Korea

**Keywords:** Paraprobiotics, inactivated (non-viable), health benefits, technological feasibilities, biological response modifier

## Abstract

Viability plays an important role in the beneficial microbes (probiotics) to produce health benefits. However, this idea has been changed after the invention of the term "paraprobiotics," indicating that non-viable microbes could produce health benefits similar to those produced by live probiotics. Occasionally, it might be dangerous to administer live probiotics to people with weak immunity. In such cases, ingestion of paraprobiotics could be a potential alternative. The definition of paraprobiotics refers to the use of inactivated (non-viable) microbial cells or cell fractions to provide health benefits to the consumer. Paraprobiotics have attracted much attention because of their long shelf life, safety, and beneficial effects, such as modulation of immunity, modification of biological responses, reduction of cholesterol, anti-inflammatory, and antiproliferative properties. These features indicate that paraprobiotics may play a vital role in improving the health of the consumer by enhancing particular physiological functions, even though the exact underlying mechanisms have not yet been completely elucidated. In this mini-review, we briefly discuss the historical backgrounds of paraprobiotics and evidence of their health-promoting effects, prophylactic, and therapeutic properties.

## Introduction

Even though it is wellk nown that probiotics have numerous health benefits, concerns have been raised about the functionality and practical use of such live microorganisms; for example, the viability of probiotic species in feed or food products, their different colonizing patterns and persistence, and the possibility of horizontal gene transfer of a virulence gene from a pathogenic bacteria in the intestine. These concerns and recent studies have proven that even non-viable microorganisms could be beneficial to consumers in a manner similar to their viable counterparts, and have accelerated the use of non-viable probiotic preparations, recently termed “paraprobiotics.”

The idea behind using non-viable microbes is to remove the many drawbacks associated with the administration of viable microorganisms. For instance, certain storage requirements are essential for the viability of probiotic microorganisms, because the desired viability of many probiotic microbes can be lost during storage. In probiotics-supplemented feed preparations, the relative proportion of viable and non-viable microorganisms might be varied, and the population of dead cells could be even larger than the viable cells. The ongoing safety concerns regarding the intake of live microorganism cells is increasing, and these concerns have intensified interest in the use of non-viable microbes or microbial cell extracts, as they could drastically reduce shelf-life problems, and eliminate the risks of microbial translocation and infection in the consumer [1]. These issues emphasize the need to explore alternative approaches such as paraprobiotic application. Additionally, current studies have demonstrated that inactivated probiotic microorganisms can also provide us health benefits [2].

Moreover, recent studies have proved that bacterial viability is not a vital requirement for health benefits [3]. Non-viable microbial cells may have safety advantages over live probiotics as they can reduce the risk of microbial translocation, infection, or enhanced inflammatory responses, which have been demonstrated by some probiotics in consumers [1]. Results of recent studies indicate that paraprobiotics impart health benefits to consumers through several methods, such as, modulating the immune system (compounds of the cell wall might boost the immune system), increasing adhesion to intestinal cells (which results in inhibition of pathogens), and secretion of various metabolites [2].

## New Concepts on Paraprobiotics

**Fig. 1:**
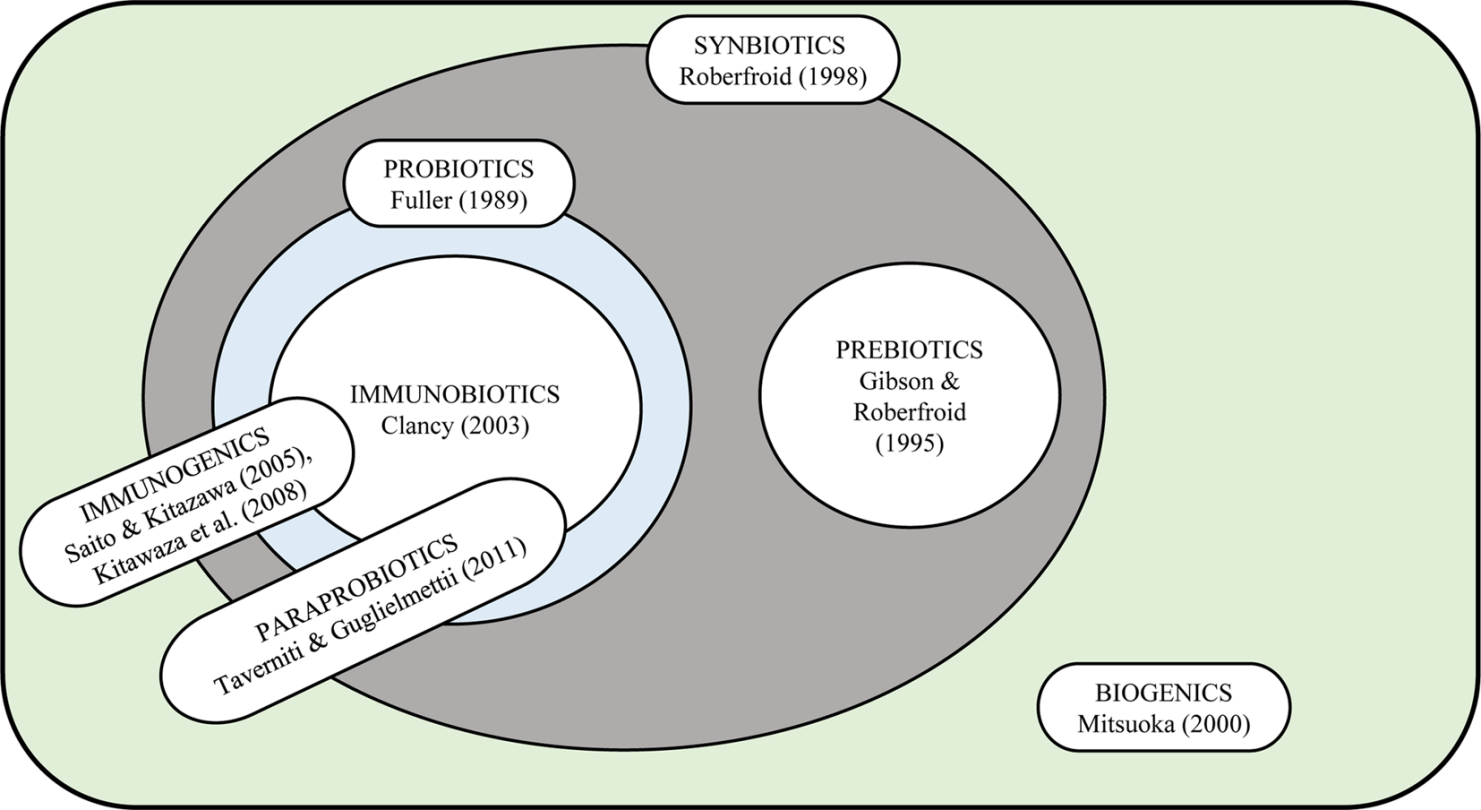
Diagrammatic representation of the interactions between the modern concepts of probiotics and related terms [5-7].

Current scientific evidence has indicated that inactivated or non-viable (dead) microbes could provide beneficial health effects to consumers. Thus, the term “paraprobiotics” has been introduced to “indicate the use of inactivated microbial cells or cell fractions to confer a health benefit to the consumer” [1]. Paraprobiotics have been previously mentioned in literature as “inactivated probiotics” and “ghost probiotics” [2]. Paraprobiotics are non-viable cells of microbes, which could be intact or broken and/or crude extracts of cells, and could produce a positive effect on the consumer when administered in sufficient amounts [1]. Recent results suggest that probiotics may exert health benefits even when they are dead (Fig. 1). This phenomenon has been labeled “the probiotic paradox” [4] and is likely explained by bioactive compounds that are released when bacterial cells dissolve in the digestive system.

Preparing a probiotic product with live probiotic microbes is still challenging. The food products first undergo thermal processing, following which the live microbes are added to the products. However, adding the live microbes to the heat sterile products can unexpectedly induce contamination [2]. From the viewpoint of technological feasibilities, the advantage of using inactivated probiotic microbes is that there is no chance of such contamination, as the inactivated microbes can be added to the food products before thermal processing. Non-viable materials of microbial origin exert marked benefits compared to probiotics for safer and more stable product development [4, 8].

## Health-Promoting Benefits, Prophylactic, and Therapeutic Properties of Paraprobiotics

Various beneficial effects, which are originated from the living cells of probiotics can also be derived from the dead cells; indeed many reports have confirmed a variety of biological responses obtained from the consumption of heat-inactivated probiotic strains. The biological response-modifying activity of dead probiotic cells is clearly similar to the oral administration of an immunization vaccine. In calves, the use of live *Salmonella typhimurium* vaccine conferred excellent protection against a challenge infection by a virulent *S. typhimurium*. Enterotoxigenic *E. coli* cells were killed by formalin treatment, and the killed cells were able to induce a strong immune response when they were used as a vaccine in human subjects. Similarly, it was observed that oral administration of inactivated whole-cell *Pseudomonas aeruginosa* vaccine was safe for human participants.

**Fig. 2:**
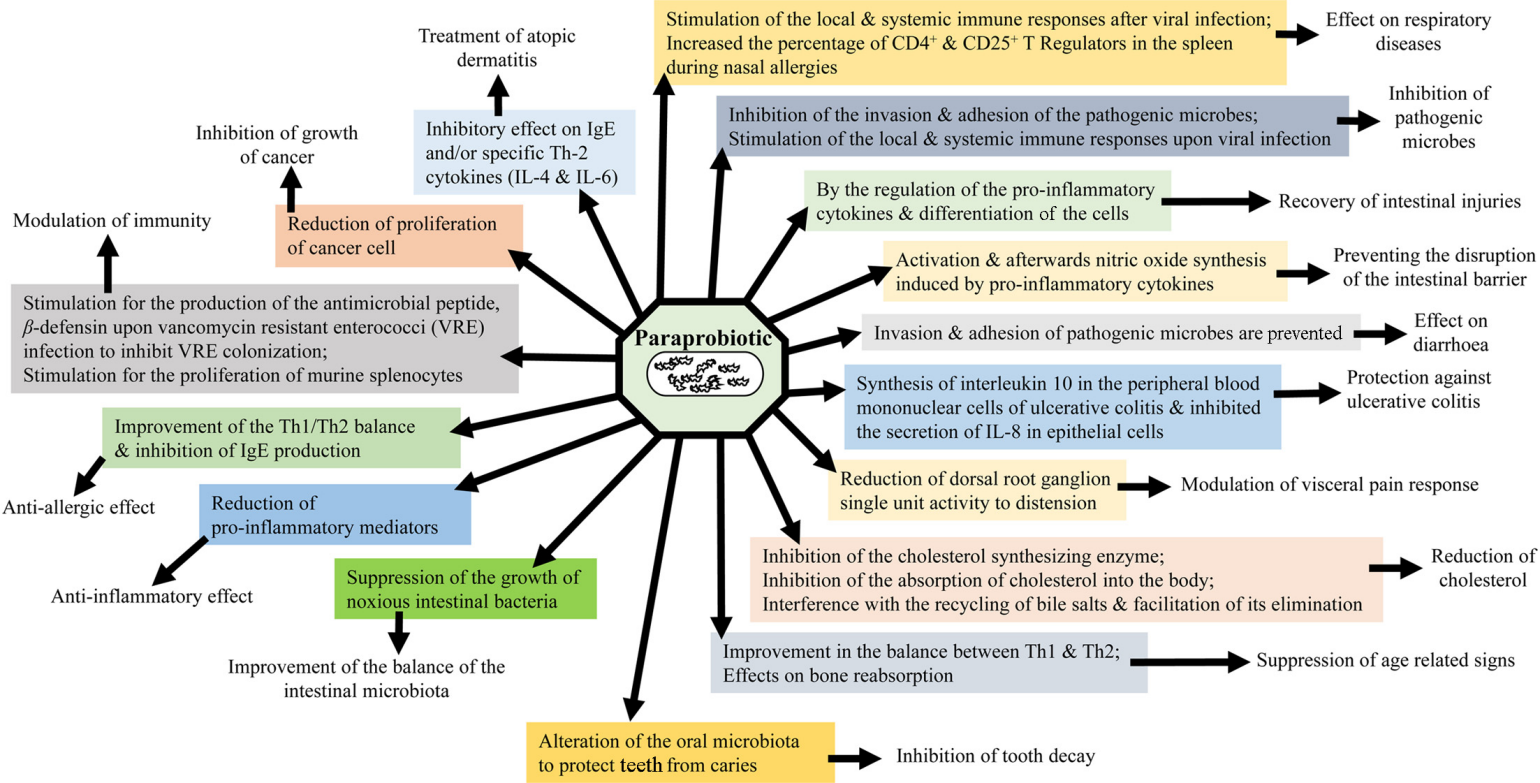
The health-promoting benefits, prophylactic, and therapeutic properties of paraprobiotics along with their possible mechanisms are shown in Fig. 2 [2, 4, 11].

Research has shown that products composed of both viable and non-viable cells can produce beneficial biological responses [9, 10]. To enhance immune responses (immunomodulatory activities), paraprobiotics which are made from heat-inactivated cells can be used. The components of dead cells display an anti-inflammatory response in the gastrointestinal tract (GIT). Notably, specific actions are exerted by both live and dead probiotics. Although the function-mechanisms of paraprobiotics are not properly clear, some literature has suggested possible mechanisms of action for their beneficial effects. The health-promoting benefits, prophylactic, and therapeutic properties of paraprobiotics along with their possible mechanisms are shown in Fig. 2 [2, 4, 11].

## Bioactive Substances of Paraprobiotics

**Fig. 3:**
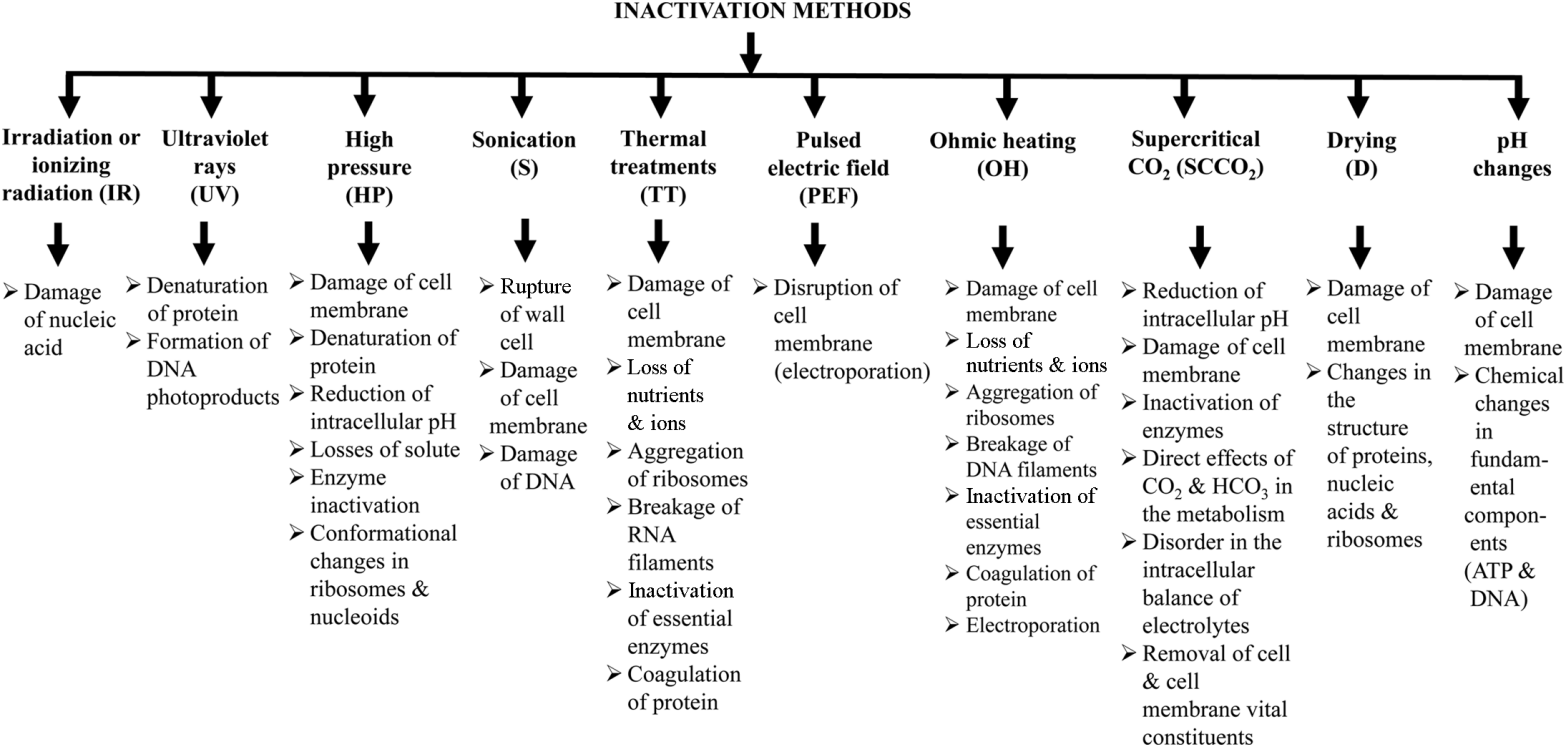
Inactivation methods of microorganisms and the underlying mechanisms [2].

Scientific evidence has demonstrated that the biogenic and paraprobiotic functions of inactivated cells of microbes, microbial fractions, or cell lysates could sustain human as well as animal health [1, 12]. On the other hand, the inactivating method would be critical to correctly interpret these results. The inactivating method (Fig. 3) can disrupt the bacterial cells, and allow for the potential interaction of intracellular bioactive compounds with the host cells on administration. Different mechanistic studies have been conducted, and their results revealed that a specific chemical compound is required to induce a specific immune response, and this compound has been isolated from microbes. Dried or fragmented cells of yeast probiotics could exert probiotic activity, and produce a beneficial effect [13]. These products are primarily composed of cell wall fragments, which contain *β*-(1,3)-D-glucans, *β*-(1,6)-*D*-glucans, chitin, and mannoproteins [1, 6]. Several other studies suggested that different components of the microbial cell, for instance, *β*-glucans, teichoic and lipoteichoic acids [14], cell homogenates [15], peptidoglycans (PGN), lipopolysaccharides (LPS), and DNA [16, 17] can produce immunomodulating effects [18-21]. The functions of bioactive compounds from inactivated probiotic cells are summarized in Table 1.

## Current Situation and Emerging Future Prospects of Paraprobiotics

The development of paraprobiotics as supplements and their application in foods and beverages comprise an important alternative to specific cases where probiotics are damaged, and not alive during processing and/or shelf life. Thus, paraprobiotics will have several applications where the addition of probiotics should involve a technological challenge. Indeed, pararpobiotic products allow for the generation of safer and more stable products over those of the live probiotic products. Therefore, the popularity of paraprobiotics is fast increasing, and in the near future, paraprobiotics will be extensively used in food, medicine, supplements, and fodder [1].

Recent studies have demonstrated that the biogenic and paraprobiotic function of dead cells, microbial fractions, or cell lysates can conserve host health [1, 12, 31, 32]. Probiotic products prepared by using dead cells could be stored without a refrigerator, and could reduce microbial translocation risk, increased inflammatory response or infection risk in consumers [33]. Serin and Andruskiene [34] reviewed a new perspective on the functional component of foods, or so-called paraprobiotics. Nevertheless, more clinical and epidemiological studies are required to understand the role of paraprobiotics on human health.

The selection of probiotic species and strains to be used for paraprobiotic production [2], the use of appropriate methods for inactivation and delivery, the evaluation of their stability and activity in foods during shelf life, and the use of adequate methods to assess their biological effects are especially important [2]. The discovery of clear potentially beneficial immunological effects of inactivated bacteria suggests that further investigation of their mechanisms of action is required. The effects of these cell wall components, after heat treatment, on the immunostimulatory activity of LAB still needs further study. Furthermore, studies should be conducted to understand the mechanism of action used by dead cells to produce beneficial effects, and whether that mechanism is similar to that of viable cells or not. Specific binding efficiencies of probiotics, as well as paraprobiotics against infectious agents *in vivo*, should be analyzed.

## Figures and Tables

**Table 1:** The summary of the functions of bioactive compounds from inactivated probiotic cells (microorganisms).

Inactivated probiotic cells (Microorganisms)	Bioactive compound(s)	Function (obtained health benefits)	Reference(s)
*Bifidobacterium lactis* Bb12	Peptides and proteins	Immunostimulating activity	[22]
*Bifidobacterium longum*	*β*-glycan	Cholesterol reduction	[2]
*Lactobacillus gasseri* OLL2716	AT motif of DNA	Enhancement of immune responses	[23]
Lactic acid bacteria	Peptidoglycan	Stimulation of immunocompetent cells	[24]
Lactic acid bacteria	Peptidoglycan	Alleviating allergic diseases	[25]
Lactic acid bacteria	Protein	Production of down-regulatory signals for peripheral blood mononuclear cell	[26]
Lactic acid bacteria	Peptidoglycans, lipopolysaccharides and DNA	Production of immunomodulating effect	[16]
Gram-positive bacteria	Peptidoglycan and lipoteichoic acid	Activation of innate immune systems	[20, 21]
Gram-positive bacteria	Lipoteichoic acids	Immunostimulatory effects	[27]
Yeast	Cell wall fragments such as *β*-(1,3)-*D*-glucans, *β*-(1,6)-*D*-glucans, chitin, and mannoproteins	Effects on ileal and total tract nutrient digestibility	[13]
*Saccharomyces cerevisiae*	*β*-glucan	Trigger the immune system	[4]
*Lactobacillus delbrueckii* subsp. *bulgaricus* OLL1073R-1	Extracellular polysaccharides	Immunomodulatory effects	[28]
*Lactobacillus brevis* KB290	Cell-bound exopolysaccharides	Enhancement of the cytotoxic activity	[29]
*Lactobacillus* strains	Lipoteichoic acids	Stimulation of innate immune responses	[14]
*Lactobacillus rhamnosus* GG	Lipoteichoic acid and peptidoglycan	Modulation of inflammation	[2]
*Lactobacillus brevis* SBC8803	Proteins	Recovery of intestinal injuries	[2]
*Lactococcus lactis* H61	Peichoic acid and lipoteichoic acid	Stimulation of vertebrate host immunity	[30]
